# Supplementary material for: A mixed methods process evaluation of the implementation of a peer coaching intervention to improve the execution of preventive tasks by occupational physicians
Source: BMC Health Serv Res. 2025 May 30;25:776. doi: 10.1186/s12913-025-12950-z (PMC12123882; doi:10.1186/s12913-025-12950-z)
Supplement: Supplementary file 1 — Supplementary Material 1. [file 12913_2025_12950_MOESM1_ESM.docx]

# Appendix 1: Questionnaires and interview guide

**Process questionnaire occupational physicians**

1. Which of the following meetings did you attend; 1, 2, 3? (Multiple choice).
2. If you did not attend one or more sessions, can you explain why not? (Open-ended question)
3. On a scale of 1-10, what rating would you give:

*• Meeting 1*

*• Meeting 2*

*• Meeting 3*

*• The guide with tips and assignments?*

*• The role of the chairperson/facilitator of your group?*

*• The intervention program as a whole?*

1. To what extent do you agree with the following statements? (Five-point Likert scale; ranging from strongly disagree to strongly agree)

*• The intervention program aligned well with my aim to dedicate more time to the execution of preventive tasks.*

*• The program helped to discuss experienced barriers and possible solutions regarding the execution of preventive tasks within my peer group.*

*• Developing a personal action plan with set goals helped me to pay more attention and time to performing preventive tasks.*

1. What goal or goals did you set for yourself during this study to focus more on prevention? Were you able to achieve this goal or these goals? (Open-ended question)
2. If you could give advice to fellow occupational physicians to focus more on prevention, what would it be? (Open-ended question)
3. What did you find good/positive about the program? (Open-ended question)
4. What could be improved about the program? (Open-ended question)
5. Space for further explanation, comments, or questions. (Open-ended question)
6. Would you like to participate in an interview? Yes/No

# **Process questionnaire group facilitators**

1. Which meetings have taken place within your group as part of the program; 1, 2, 3? (Multiple choice)
2. If not all 3 meetings have taken place, can you describe the reason? (Open-ended question)
3. To what extent were you able to achieve the following aspects? (Five-point Likert scale, ranging from “not achieved at all” to “fully achieved”)

*• Were you able to discuss the wishes and challenges regarding preventive tasks with the members of your group?*

*• Were you able to discuss possible solutions for the experienced barriers among the members of your group?*

*• Were you able to engage the members of your group and get them actively involved in the intervention?*

1. On a scale of 1-10, what rating would you give:

*• Meeting 1*

*• Meeting 2*

*• Meeting 3*

*• The guide with tips and assignments?*

*• Your role as chairperson?*

*• The online training for chairpersons/facilitators?*

*• The instructions for chairpersons/facilitators (manual, agenda, PowerPoint slides)?*

*• The intervention program as a whole?*

1. What goal or goals did you set for yourself during this study to focus more on prevention? Were you able to achieve this goal or these goals? (Open-ended question)
2. If you could give advice to fellow occupational physicians to focus more on prevention, what would it be? (Open-ended question)
3. What did you find good about the program?
4. What could be improved in the program?
5. Space for further explanation, comments, or questions.
6. Would you like to participate in an online interview? Yes/No

# **Interview guide**

1. What do you think of when we talk about “preventive tasks” or “prevention”?
   1. In what way were you already engaged in performing preventive tasks in your work before participating in this study?
2. For what reason or expectation did you (with your group) sign up for participation?
   1. What sparked your interest?
   2. To what extent did the program in the ICT groups meet your expectations?
3. What goal did you formulate to work on during the program's duration?
   1. Why did you choose this goal?
   2. Were you able to achieve this goal or these goals? What was successful and what was not, and why? Can you tell us more about how you approached this?
   3. What barriers did you encounter earlier? Has your participation in the study changed anything in this regard?
   4. What advices did you receive from your ICT group/colleagues? Were these advices helpful and why?
4. What did you find most appealing about the program and why?
5. What did you find least appealing about the program and why? How could we improve this?
6. Would you recommend this program to other occupational physicians (and groups) who want to focus more on prevention? Why or why not?
7. Are there other ways to better integrate preventive tasks into the work of the occupational physician or to put the topic of prevention more on the agenda?
8. We find sharing good experiences/best practices very important. Therefore, we would finally like to ask you: If you could give one piece of advice or share an experience with fellow occupational physicians to focus more on prevention, what would it be?

**Additional questions for group facilitators**

1. Below are a number of statements. Can you indicate to what extent you agree with these statements?
   1. *Procedural clarity: The intervention program clearly describes the activities I should perform and in which order.*
      1. Was the provided guidance (training/contact/manual) sufficient to prepare you for the meetings? Why or why not? What, if anything, did you miss?
   2. *Completeness: The intervention program provides all the information and materials needed to work with it properly.*
      1. Did you miss anything?
   3. *Complexity: The intervention program is too complex for me to use.*
      1. Can you elaborate on this? What was successful? Were there things that did not work, or did you encounter any barriers?
   4. *Compatibility: The intervention program is a good match for how I am used to working.*
      1. Do you think this is the most suitable working method? Why or why not? Are there alternatives that would have worked better for you?
   5. *Relevance: I think the intervention program is relevant for my peer group.*
